# Accounting for the Unaccounted in AI and Care: Bringing Young Carers into the AI Realm

**DOI:** 10.2196/83021

**Published:** 2026-10-09

**Authors:** Laetitia Tanqueray, Chris Papadopoulos

**Affiliations:** 1 Department of Technology and Society, Faculty of Engineering, Lund University, Klas Anshelms väg 14, Lund, Sweden, 46 462229129; 2 Independent Researcher, London, United Kingdom

**Keywords:** young carers, artificial intelligence, care, informal care, norms-in-the-loop, socio-legal, public health, children’s rights, participatory design methods

## Abstract

This position paper introduces young carers as a key stakeholder within AI. Research on AI for health care endeavors to assist and optimize current health care services; yet, young carers have been neglected thus far. Accordingly, this paper situates itself at the intersection of young people under the age of 18 years old providing care (referred to as young carers) and AI research. Through a socio-legal and public health lens, this paper (1) situates young carers to showcase (2) why they must be accounted for in AI systems, and (3) how AI researchers can do so. This lens highlights the difficulties young carers tend to face at present and how AI systems can alleviate those—rather than reproduce them. Accordingly, we rely on the norms-in-the-loop framework to demonstrate why the AI community must account for young carers in AI systems. We first synthesize research on both young carers and AI research on contextual, legal, and social norms to then propose practical ways to do so through design, datasets, and in situ personalization. Overall, this paper offers a critical foundation for AI researchers to include young carers in the development of AI systems at various levels: from institutions to care receivers, and to young carers themselves. Ultimately, this paper posits that young carers must be identified and supported, which demands their inclusion in AI research to understand how best to do so. Accounting for young carers will allow for their unique perspectives to be reflected in AI systems and help them thrive beyond their care responsibilities.

## Introduction

*“[Young carers] experience a lifetime of coming second (or third, or fourth) to the needs of another.” *[1]

This quote is from the United Kingdom’s All-Party Parliamentary Group (APPG) for young carers report, to showcase the harsh realities children providing care face. The APPG report [1] represents the first ever UK Parliament inquiry into young carers—released in November 2023. This report was an urgent call for action to better attend to young carers’ needs through identification and targeted support. Young carer is a term used to refer to children under the age of 18 providing care for a relative or friend and is usually a hidden form of care in society. Although, importantly, it is estimated that 6%-8% of the child population are young carers [1-3] and less than a handful of studies have included young carers in AI research thus far.

Research in AI is attempting to understand how to deploy technologies that can assist in education [4], health [5], and everyday tasks [6]. While AI researchers include children in studies to understand their perspective, this has seldom been done with children in their role as young carers [7]. This paper argues that we, the AI community, must attend to the caregiving role children do: young carers’ context and perspectives must be accounted for in AI systems since they are and will be impacted by these technologies. Furthermore, by accounting for young carers, AI systems would in part allow for (earlier) identification—especially identification of excessive care responsibilities—as well as support for children who have to provide care.

From a medical informatics perspective, young carers raise questions about data representation, stakeholder modeling, proxy variables, workflow integration, and the governance of AI-generated alerts. Their caring role may be medically and socially relevant, but it is often absent from structured data, inconsistently recorded across institutions, or known only informally by professionals [8]. This creates a risk that AI systems will either ignore young carers entirely or infer caring responsibilities from indirect and potentially misleading indicators. The challenge is therefore not simply to add young carers as another user group, but to determine when young carer status is relevant, how it can be represented without exposing children to harm, and how AI outputs should be embedded into professional workflows that provide support rather than surveillance.

This position paper directly situates itself at the intersection of young carers—especially in the United Kingdom—and AI. The United Kingdom provides a concrete case with clear regulation on young carers [2,3], which can serve as an initial model of good practice for other countries. Through the norms-in-the-loop by Larsson et al [9], a socio-legal and public health lens is used to highlight the challenges ahead and explore ways to transform them into opportunities for the AI community. *AI community* here is used as an all-encompassing community varying from researchers in human-computer interaction (HCI), human-robot interaction (HRI) to digital health and medical informatics, to projects investigating uses of machine learning, foundation models, personalized conversational agents, or smart assistants. *AI* represents a field of development and inquiry which takes the form of various technologies, such as smartphone apps and social robots. In this paper, we define *AI systems* according to the definition by UNICEF (United Nations International Children’s Fund), whereby AI systems make predictions, recommendations, or decisions that influence real or virtual environments according to objectives defined by humans [10]. Importantly, we use three different terminologies concerning AI in this paper, which are: (1) AI systems for general claims; (2) AI research to showcase what research is being done in this area and how it could include young carers; and (3) the specific modality where the point only applies to, for example, predictive systems, social robots, conversational agents, or educational analytics.

The novel contribution of this position paper is threefold. First, it identifies young carers as a distinct and currently underspecified stakeholder group in AI and digital health research, rather than treating them as an incidental part of the household or as a subgroup of adult informal caregivers. Second, it applies the norms-in-the-loop framework [9] to show how the invisibility of young carers can become embedded across AI systems’ design, dataset construction, and in situ personalization. Third, it translates this socio-legal and public health analysis into practical design and governance implications for AI systems that may affect young carers directly or indirectly. In doing so, the paper moves beyond a general call for inclusion and argues that accounting for young carers is an informatics, ethical, and children’s rights issue; AI systems that do not recognize the caring role of children may reproduce institutional blind spots [11-13], misclassify family situations, increase surveillance risks [14,15], or place additional responsibilities on already overburdened young people [1].

## Methodological Approach

This position paper is based on a narrative and conceptual synthesis rather than a systematic review. Its purpose is not to provide an exhaustive account of all AI, young carer, or informal caregiving literature, but to integrate the following three bodies of work that are rarely brought together: (1) socio-legal and public health lens, (2) young carer research, and (3) AI research. Relevant literature was identified through the authors’ previous work on informal caregivers’ involvement in human-robot interaction studies [16], as well as young carers and AI [7]. These studies demonstrated an oversight of young carers in AI research, which necessitates targeted searches of literature on young carers (including academic scholarship as well as State-level reports), informal caregiving technologies, participatory design, social robotics, digital health, and AI ethics, and key legal and policy documents relevant to children, carers, data protection, and AI.

Sources were selected for their relevance to the paper’s central conceptual question: how might AI systems reproduce, challenge, or transform the invisibility of young carers? The young carer evidence base was anchored in the APPG report [1], cross-national analyses of young carer policy and recognition [2,3], and an overview of young carer research, practice, and future priorities [17]. The AI and digital health evidence base drew on reviews examining informal caregiver inclusion and AI-supported care [16,18-20], supplemented by international child-rights instruments and policy guidance concerning children’s participation in AI and digital environments [10,21]. Targeted searching was supplemented by backward and forward citation searching from these foundational sources. The resulting literature was then synthesized deductively using the contextual, legal, and social norms categories within the norms-in-the-loop framework by Larsson et al [9].

The synthesis was guided by the norms-in-the-loop framework by Larsson et al [9], which distinguishes between 3 types of norms embedded in design, datasets, and in situ personalization in AI systems. We use this framework as an analytical structure to examine how contextual, legal, and social norms surrounding young carers as their norms will become embedded in AI systems even if not accounted for. The resulting recommendations should therefore be read as conceptual and practice-oriented propositions intended to guide future empirical, participatory, and technical research, rather than as findings from a systematic evidence review.

*Norms-in-the-loop* [9] argues that norms—namely contextual, legal, and social—must be considered, as they form the foundation, or *expectations*, of AI systems; this includes deployed AI systems to those still at the design stage. These norms are inherently intertwined in AI systems’ design, datasets, and in situ personalization (Figure 1). Put differently, norms are at the core of AI systems, and those norms should be accounted for so as not to perpetuate problematic societal issues. In this paper, we begin with contextual norms, to present and situate both young carers and AI research in health care. Followed by legal norms, which are specific legal rules on young carers, children, and AI, data protection, and data privacy. Finally, social norms are presented to showcase that despite laws in place, there are issues around identification and support for young carers and an exclusion of young carers in AI research. We then use those to present strategies on AI systems—referred to as *adaptive technologies* in work by Larsson et al [9]—to directly showcase how to account for young carers in practical terms. These are design, datasets, and in situ personalization. Ultimately, by accounting for young carers in those categories, we premise that there would be a better possibility to identify and support children in their care roles, although for now this possibility is neglected. We also specifically leverage on AI community’s research practices around participatory design to demonstrate why this method is required in such a sensitive area.

**Figure 1.**
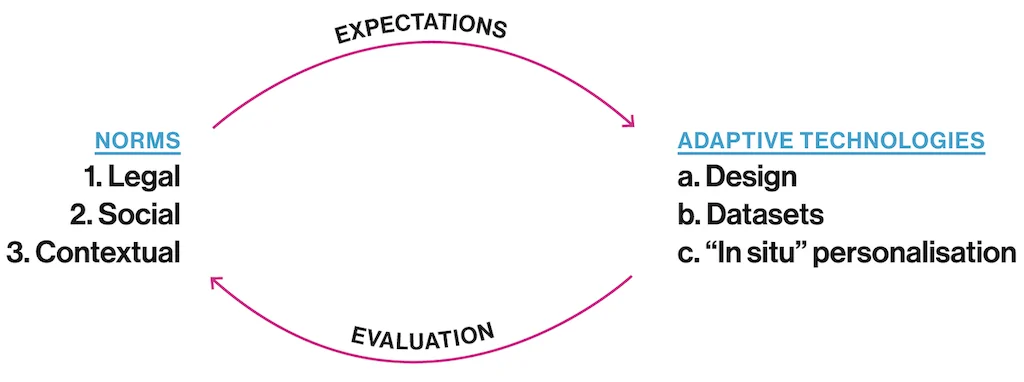
Theoretical framework “norms-in-the-loop”: the mirroring of norms (reproduced from) by Larsson et al [9]*.*

It is important to note an important historical critique from disability rights scholars around young carer service design, as these can undermine the parental role of the parent with a disability [22,23]. These debates took place in the 1990s and showed a conflict between children’s rights approach versus a disability rights approach; the first primarily aimed to empower children, while the other primarily aimed to empower the person with disabilities without pathologizing their disability [23]. Ultimately, children’s rights scholars—also young carer scholars—argued that while services have to be available for persons with disabilities, children are still providing care and must also have access to services to support them [24]. Although this is a historical debate, AI researchers should be aware of it to ensure that as a community we adopt an inclusive approach that considers the broader family dynamics as well as the young carer. Therefore, while we fully support the need to ensure that care receivers access and receive adequate care, we aim to show why it is essential to research and design AI systems beyond care receivers and account for their family setup. For example, predictive systems on care receivers’ care needs that do not account for young carers could reproduce their current overlook and responsibilities, and thus their care load would not be reduced. By taking a whole-family approach [25], we can ensure that these AI systems are beneficial and effective for everyone involved, which has to include informal caregivers. Furthermore, while informal caregivers usually provide essential care to the person they are providing care for, *young carers are children and young people first* [26]. This position paper therefore serves to advocate that young carers should be recognized as a vital user-group, and AI systems should ensure that they are also designed for them as carers as well as young people.

## Contextual Norms: Bringing Awareness Around Young

### Overview

Larsson et al [9] argue that questions of fairness need to be contextualized and situated when a practice becomes intertwined with technology. This is in direct contrast to universalizability (of care, in this instance). This is key in this paper as we try to situate an informal care practice that has been mostly overlooked by the AI community and thus requires contextualizing. Accordingly, this section aims to showcase the context of young carers, as well as a sample of AI research on informal caregivers.

### Situating Young Carers

Beyond AI research, young carers have been well-researched within law and social sciences. Various studies have consistently indicated that between 6% and 8% of children in advanced industrialized capitalist societies are carers [2,3]. However, these estimates are based on young carers known to authorities, meaning that the actual numbers are likely even higher [1-3]. Although there is no clear definition of young carers, we use Becker’s [27] widely used definition by the young carer research community and practitioners:

*Children and young persons under 18 who provide or intend to provide care, assistance or support to another family member. They carry out, often on a regular basis, significant or substantial caring tasks and assume a level of responsibility that would usually be associated with an adult. The person receiving care is often a parent but can be a sibling, grandparent or other relative who is disabled, has a chronic illness, mental health problem or other condition connected with a need for care, support or supervision.* [27]

This conceptualization demonstrates that young carers are (1) under 18 years of age, (2) providing a form of care, (3) to someone that can be either a family member (ie, parent, grandparent, and sibling) or a friend, (4) care often happens on a regular basis. Beyond this definition, we add that (5) caregiving has positive and negative impacts on young carers [28], (6) young carers fall under the broader category of informal caregiving, meaning that despite the demands of their care role, it is unpaid and no training is provided, as well as (7) young carers are typically demographically very close to the person they care for, whereby the care receivers and young carer usually live in the same household.

Becker [29] has illustrated young carers’ responsibilities on a caregiving continuum, ranging from “caring about” to “caring for,” which can be gradual for a young person providing care (Figure 2). This demonstrates that household chores can be part of caregiving; however, it is also likely to be part of everyday life for a child who does not provide care. As Figure 2 illustrates, the task together with the level of responsibilities are what differentiates between a child that provides care and one that does not. Although there is not a one-size-fits-all description of tasks young carers do, these usually include one or more of the following: general domestic tasks (such as cleaning), household management, finances, sibling care, acting as the translator for the care receiver, providing direct care (such as emotional support and supervision), personal and intimate care, health and medical care, and self-care for the young person to ensure their own well-being [2,30,31].

**Figure 2.**
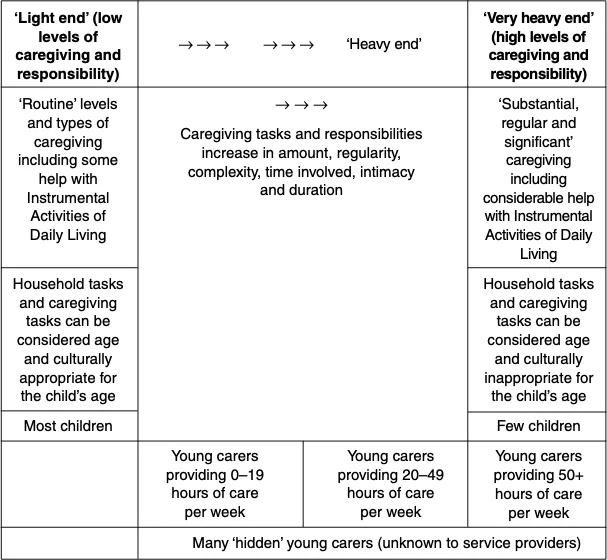
Becker’s illustration of “A continuum of children’s caregiving” (reproduced from Becker [29]). The arrows pointing to the right represent an increase in care responsibilities.

Similarly to other groups of informal caregivers, young carers provide care and support for various conditions and types of care receivers. The most extensively researched subgroup of young carers is those who take care of a parent. Those young carers might be caring for a parent due to substance abuse, chronic illness, disability, or mental health issues, among other factors [32,33]. Another subgroup of young carers provides care to a sibling due to disability (such as autism) or chronic illness (such as cancer). This does not eliminate the role of the parent(s), although some evidence suggests that young carers providing care for siblings take on more care responsibilities than young carers taking care of a parent [34]; yet there is a lack of awareness on how much responsibility they take on—including by their parents [35]. Another subgroup of young carers takes care of a grandparent, due to a disability or chronic illness, such as dementia, although not as much research has been conducted on this. One study suggests that there is a lack of clear identification for this subgroup, in part due to their responsibilities being somewhat more occasional and that the young person is often not the primary carer [36]. Finally, there is a group of young carers emerging in research around young people providing care for their friends, due to disability, mental ill-health, or chronic illness; 1 study showed that this segment of young carers reported more health problems—especially with regards to mental health [37].

The impact of caring is also an important indicator to account for. Aldridge [28] highlighted that research suggests that the impact of providing care can be dependent on the duration of care; for example, a young person caring for someone for longer than 2 years is more likely to experience mental health issues. Put differently, if the impact of caregiving is major on the young carer, the regularity on the caregiving continuum might not be as useful. Nonetheless, caregiving can have a positive impact on the young person. Cassidy et al [38] demonstrated that there is increased resilience in young people who provide care, as long as it is not overly excessive *and* is socially recognized. Leu et al [3] accentuate current research on the positive impacts of young people caregiving, which include increased maturity, closer relationships to parents, feeling more prepared for life, as well as being more empathetic and compassionate. Although there are also negative impacts, such as higher absenteeism and dropout rates from school [1], as well as anxiety [39], depression [40], and social exclusion [41]. In the most recent report on Not in Education, Employment or Training (NEET), in June 2026, young carers were “2.2 times more likely to be persistently in the NEET category for two or more years” [42], demonstrating that providing care can significantly impact young people’s lives into adulthood.

The UK context is also pivotal here, as young carers have a clear legal status and research base on young carers is well-established [2,3]. This results in UK institutions reporting on young carers [42,43] to pinpoint how to better support them [1]. This is in direct contrast to all other countries that do not have clear regulations on young carers and thus lack research and understanding on how to operationally support young carers [2,3]. Briefly contextualizing the specific and complex UK socioeconomic setting, Vizard et al [8] demonstrated that the poverty rate among young carers is highest in comparison with other groups of children, and also found increasing rates of poverty in young carers’ households. Moreover, while key characteristics of young carers were recorded, they were often not separately analyzed in public statistics, or they were underrepresented due to small sample sizes, leading to their exclusion. The report also pointed out that before the 2008 global financial crisis, young carers tended to be protected from poverty, demonstrating worsening welfare provisions [8]. Furthermore, other studies have suggested that families of young carers in need of care do not receive sufficient publicly provided care [44,45]. This has been exacerbated by diminishing availability of formal support services generally across countries [46], and worsened by the COVID-19 pandemic, with the lack of public services available leading to more care responsibilities being performed by young carers [47]. This increase in young carers has led to the Department of Education requiring schools to report on the number of young carers [43], as well as specifically accounting for young carers when looking at the NEET population [42]. These insights demonstrate how young carers can become the *default* carer due to lack of support and services available for formal carers in the United Kingdom to take on care responsibilities, with repercussions on their future. The data on young carers in the United Kingdom showcases why more research needs to be done to understand young carers’ context in different countries for AI systems to alleviate their care responsibilities.

### Informal Caregivers and AI

Much AI research aims to relieve pressure and/or provide support for specific user groups. Al Kuwaiti et al [18] reviewed AI systems in health care generally. They demonstrated how revolutionary AI has been and will continue to become, including in virtual patient care, patient engagement and compliance, rehabilitation, and other administrative applications. Although, we add, that these applications have to acknowledge informal caregivers and AI systems must dynamically adapt to changes in caregiving over time [48]. Therefore, this section highlights the AI community’s capacity to include young carers as a distinct user group, especially given that adult informal caregivers are already somewhat considered as such.

Schinkinger and Tellioğlu [49] published design implications for technologies specifically to support (adult) informal caregivers in their daily role. They provided qualities and features that technologies should have to meet informal caregivers’ requirements, which included communicating with professional care services, self-help groups, and monitoring care receivers. In a recent scoping review, Premanandan et al [20] outlined available technologies for informal caregivers. Their findings point out that informal caregivers can use technologies to optimize their information access, such as relevant information related to caregiving, as well as access social support to feel less alone in their situation. Although there were also barriers, which related in part to privacy concerns, lack of trust in these applications, and lack of time to use these applications to name a few. Borna et al [19] systematically reviewed AI support for informal caregivers which showed promising results to provide smart, adaptive support, improving carers’ effectiveness and well-being which could help with care responsibilities. Nittas et al [50] conducted a scoping review on the use of digital technologies for family care, where they found reports on psychological well-being, carer competence, quality of life, carer-patient relationships, as well as care coordination and efficiency. Although they concluded that additional research with more inclusive and person-centered digital informal care approaches are needed—highlighted by their findings that out of 110 studies, only 39 included informal caregivers, patients, and/or other stakeholders in their design.

Messina et al [51] investigated online interventions that can provide support and self-learning tools specifically for informal caregivers who provided care for a person living with dementia, named “iSupport” (developed by the World Health Organization). This platform has also consulted young carers directly so that the smartphone app is also accessible to them and is designed in a way that reflects their own reality (ie, illustrations of children caring, not just adults) [52]. Although Messina et al [51] found that there was a reluctance to use iSupport on behalf of (adult) informal caregivers as it created a level of burden, a sense of duty to use it, fear of being misunderstood by others, as well as difficulty in reaching relevant information. Khalid et al [53] investigated the use of mobilize digital carer support system for informal caregivers, a platform which consists of the carer’s assessment tool and a mobilize assistant chat. The latter uses supervised machine learning and retrieval-augmented generation to respond to informal caregivers’ queries, based off other peer-generated content. From this study, Khalid et al [53] found increased satisfaction among (adult) informal caregivers, reducing social isolation and enhancing accessibility, inclusion, and quality of support. Hanson et al [54] included young carers, in part to create a smartphone app that met their needs, with the aim to provide psychosocial support to promote the mental health and well-being among adolescent young carers (although not through AI systems).

Social robots, robots that interact and communicate with humans [55], are also being developed for health care purposes [16]. Although the literature review by Tanqueray et al [16] found that most studies in HRI for health care did not tend to include informal caregivers. Laban et al [56] investigated the deployment of a social robot to elicit self-disclosure for informal caregivers over 5 weeks. The preliminary findings were positive and found that informal caregivers opened more about their feelings over time, which helped alleviate some of their concerns in caregiving. Amabili et al [57] investigated the use of eWare, lifestyle-monitoring technology within a social robot, in order to reduce stress and improve quality of life for both informal caregivers as well as people living with dementia. Within their findings, they stated that “the impact of the system in reducing the carers’ burden needs to be deeply investigated” [57]. Lee et al [58] explored the views of care receivers and their informal caregivers to demonstrate their potential conflicting views and interest that would need to be taken into account for the design of socially assistive robots; these findings were also echoed in studies by Moharana et al [59] and Winkle and Moradbakhti [60]. Tanqueray et al [7] introduced social robots to young carers through participatory methods. In this study, they found that young carers used AI systems, such as smartphones and smart voice assistants. Their findings also showed that young carers were receptive to social robots to provide care, although they expressed privacy concerns and negative impacts on resource allocation. Finally, Baji et al [61] demonstrated informal caregivers’ openness to paying for robot services to help with caregiving responsibilities, showcasing that robots are expected to be part of the health care system.

### Takeaway on Contextual Norms

This section demonstrated the current realities of being a young carer. Although young carers and care receivers cannot be categorized as a homogenous group, young carers exist and their invisible care responsibilities impact their daily lives, from school attendance to mental health. In parallel, this section also demonstrated that AI research overall lacks consideration of young carers. However, already much research has been conducted on young carers outside of AI, with various reports establishing evidence for the need for better identification and support for young carers for them to thrive. This paper thus presents a new AI research avenue that is much needed to account for young carers’ key role in society and support them.

Contextual norms demonstrate the need to situate and attend to young carers and their care practices. This is especially significant as their practices are more local than medical professionals’ since the care provided will be for close relatives or friends. As AI systems continue to be developed to render health care services (more) accessible, these technologies must account for different stakeholder groups that must include young carers and their various contexts. Examples of this include developing smartphone apps that are adapted to young people for caregiving or education, or identification tools within health care databases. This is all the more important as AI systems represent expensive technologies, especially robots, which would likely not be affordable to young carers’ families but would benefit from those. Consequently, this paper argues that young carers must be part of this process, regardless of the likelihood of young carers’ families being able to afford them, as those could be made accessible through public services.

## Legal Norms: Laws for Young Carers and AI

### Overview

Law provides a set of rules that govern society, which individuals must follow. Legal norms refer to interpretations and applications of legal texts [62]. Legal norms are instrumental to AI systems since laws shape expectations in society and in turn AI systems [9]. In this section, we first map out the significance of law for young carers—although this only applies in the United Kingdom as it is the only State to give young carers a specific legal status [3]. Nevertheless, the United Kingdom provides a clear case to demonstrate an actionable way for AI researchers to push for more recognition of young carers in AI research, beyond the United Kingdom. By highlighting specific laws and guidelines that directly affect young carers and/or uses of AI systems, we show that this signifies that young carers must be explicitly incorporated when developing AI systems designed for, and in the best interest of, young carers.

### Significance of Law for Young Carers

As mentioned above, only the United Kingdom has the most advanced legislation and policies in place for young carers [2,3]. Meaning that the United Kingdom (1) exhibits widespread awareness and recognition of young carers, (2) has laws in place for young carers, and (3) has a good research base [2].

Establishing laws for young carers symbolizes that there must be State intervention [63]. Law legitimizes that a child has care responsibilities, and that the State should intervene by identifying young carers and supporting them in their care role—if the child must provide care [30]. Other countries with strong welfare States, such as Sweden, have not established laws on young carers. Nordenfors and Melander [64] criticize Sweden on this, as it leaves children to provide care without support. Put differently, by not having laws in place, the child will still provide care but not be identified or supported by relevant institutions. Consequently, legislation for young carers provides necessary welfare provisions to ensure essential assistance and well-being measures; although as Leu and Becker [2] point out, this demands a good research base. This is well-illustrated by the APPG report, which relies heavily on national research to evidence hardships young carers face [1].

UK legislation provides a concrete case of how legal norms can require institutions to recognize, assess, and support young carers. The specific statutory duties are not directly transferable to other jurisdictions, but the underlying questions have wider relevance—whether young carers are legally or institutionally recognized; which organizations hold responsibilities to identify and support them; what services follow identification; and how children’s participation, privacy, and data rights are protected. Applying norms-in-the-loop in another setting therefore requires recalibration to local law, welfare provision, service capacity, cultural understandings of childhood and family care, available data, and the particular AI system under consideration. The UK model should not simply be exported. Instead, AI researchers should work with local young carers, practitioners, policymakers, and legal experts to determine whether, when, and how caring contexts should be represented and acted upon, with the aim of reducing excessive caring responsibilities and improving support without creating surveillance or stigma.

### Applicable Laws in the United Kingdom for Young Carers

The United Kingdom consists of the following 4 nations: England, Wales, Scotland, and Northern Ireland. In practice, this means that laws for young carers are fragmented and regulated under different national legislation which are not standardized [3], as it falls under social care provisions. All legislations pertaining to young carers in the United Kingdom aim to prevent, identify, assess, and support young carers. In Wales, young carers are regulated under the umbrella of informal caregivers [65]. In a similar vein, in Scotland, young carers are part of a carer-specific legislation [66]. Whereas in England and Northern Ireland, young carers are regulated under law pertaining to children (in England [67-69] and in Northern Ireland [70]).

These laws create a legal duty on local authorities to have appropriate services in place to identify, assess, and support children that might be (or become) young carers. Through an assessment, local authorities can put in place various arrangements to alleviate or eradicate the care load from children taking care of a relative. This can take the form of respite for young carers, by attending support groups targeted at children that provide care. Furthermore, the legal recognition of young carers allows for some available State benefits in all 4 nations, namely the *Carer’s Allowance.* However, the material support is limited; the young person *must be older than 16 years of age* and *left full-time education* to take care of the person they care for; the current rate is paid at a standard rate of 83.30 GB pounds (approximately US $110; 2025-2026) a week, and the person cannot earn more than 196 GB pounds (approximately US $260) per week after tax from another employment [71,72]. Only Scotland offers a Young Carer Grant—a lump sum of 390.25 GB pounds (approximately US $520). Meaning that any young carer between the ages of 16 to 18 years who care for more than 16 hours a week in the last 3 months can apply to the grant without it affecting their education or employment [73,74].

These national laws concretely try and operationalize on researchers’ findings, which time and again call for better identification and support for young carers, to ensure that they can thrive in life [1,42,75,76]. Although these legal norms are specific to the United Kingdom, they demonstrate how young carers could be better supported through clear legalization. Furthermore, there are also international legal instruments in place to ensure that every child can thrive—which by default includes young carers—and this is beyond the United Kingdom.

### International Legal Instruments on Children and AI

The United Nations Convention on the Rights of the Child (UNCRC) [21] and UNICEF’s policy guidance on AI and children [10] provide concrete frameworks on empowering children’s voices, in society generally and AI systems (eg, on age-appropriate AI [73]).

The UNCRC represents one of the most widely ratified Conventions by States (all States except the United States) [77]. For most States, this Convention is not legally enforceable (including the United Kingdom); however, States must follow the convention in principle of good faith [78]. The Convention is based on fundamental values represented by 4 articles including the right to nondiscrimination (Article 2), the best interest of the child (Article 3), the right to life and development (Article 6), and the right to be heard (Article 12) [21]. This is applicable to anyone under the age of 18 years old. Within the context of young carers, applicable UK institutions accountable to the UN’s Committee on the Rights of the Child have specifically undertaken work to empower young carers’ voices directly through a survey filled out by more than 6000 young carers and present their needs [79], demonstrating that young carers must have their unique perspectives accounted for—beyond the United Kingdom. Put differently, the UNCRC can be relied on by AI researchers to include and account for young carers’ perspectives.

The UN’s Committee on the Rights of the Child released a General Comment in March 2021 on the digital environment and children [80]. Within the opening paragraphs, the Committee states: “if digital inclusion is not achieved, existing inequalities are likely to increase, and new ones may arise” [80]. The Committee’s general comment iterates that (1) although the digital environment was not originally designed for children, it plays a significant role in children’s lives and therefore children’s best interest should be paramount (in line with Article 3, UNCRC) [80]. In line with this, (2) children must be supported to participate as equally as adults within this environment, and thus their views need to be considered as well (in line with Article 12, UNCRC) [80]. In November 2021, UNICEF released a policy guidance on AI and children [10], which references the general comment directly and bases itself on the UNCRC to uphold children’s human rights. Accordingly, this guidance reflects key principles within the UNCRC and directly calls out the lack of children’s involvement in current technology design and national AI strategies.

While the legal norms from the UNCRC and UNICEF’s policy guidance on AI and children provide explicit justifications to include children that provide care, collecting data on children is very sensitive and must adhere to additional domestic laws, such as privacy laws.

### Protection of Privacy

Data protection is regulated by States throughout the world to protect personal data and privacy—an issue studies on informal caregivers report as an obstacle to using AI systems [7,20]. The European Union has a comprehensive regulation on data protection, the General Data Protection Regulation (GDPR) [81]. The United Kingdom has adopted the UK Data Protection Act 2018 (DPA; section 3(10), [82]) which aligns with the GDPR. Both laws’ purpose is to provide a clear legal framework on how organizations collect, use, and protect personal data of individuals in the United Kingdom and European Union. The UK DPA maintains the same core principles as the GDPR, such as lawfulness, fairness, transparency, and data minimization. It also upholds the rights of individuals, including access to their data, the right to erasure, and protection not to be subjected to automated decision-making.

The UK’s DPA makes special provisions regarding children. For one, it lowers the age of consent to 13 years old (Article 8(1))—in comparison with 16 years old in the EU (European Union) GDPR (section 8(1)). Furthermore, Article 123 of the DPA demands age-appropriate design of Information Society Services, such as social media, so that children can understand the privacy notices and give informed consent. This has direct consequences on how AI systems can operate when capturing information, for example, through sensors, direct input from users, data mining, or third-party data feed (ie, smart watch sharing live updates to another monitoring platform). With regards to young carers specifically, this means that any AI system collecting their data will need to explain its purpose in a child-friendly manner which also clearly defines the purpose of the application so that the child (and/or guardian) can make an informed decision on using it.

The Information Commissioner’s Office (ICO), an executive body in the United Kingdom specific to data privacy, has also released “The Age Appropriate Design Code” which explains that data sharing of children’s data must be based on the UNCRC’s child’s best interest [83]. The Age Appropriate Design Code has for objective to improve quality and efficiency of services by allowing better data sharing between health care and social care institutions. Put differently, the data collection cannot be simply to optimize the algorithm, it must primarily account for the child’s best interests. We add that this concern is valid beyond the United Kingdom, and collection of data concerning young carers must follow the UNCRC.

### Takeaway on Legal Norms

This section demonstrated that informal care practices are not regulated, and that available laws in the United Kingdom aim to identify and support young carers. Young carers would benefit from such laws across the globe to support young carers to manage their care responsibilities and daily lives, as discussed in the contextual norms section. Therefore, other legal instruments can be relied on, such as the UNCRC, as AI systems are integrated into health care and education services to ensure that young carers are accounted for. For example, these systems can include educational analytics that would provide more some insights on the correlation between care responsibilities and impacts on educational attainment.

Ultimately, the legal implications are not abstract, they shape concrete design decisions—what data should be collected, whether young carer status should be directly recorded or inferred, who can access that information, when an alert should be generated, what explanation is given to the child, and what support follows. A children’s rights approach requires AI systems affecting young carers to be assessed not only for technical accuracy, but also for proportionality, explainability, privacy, safeguarding, nondiscrimination, and the child’s best interests. In practice, this means that AI systems that accurately predict possible young carer status may still be inappropriate if those disclose sensitive family information without consent, increase institutional monitoring, or route children into responses they experience as threatening rather than supportive.

## Social Norms: The Gap Between Law and Society

### Overview

Larsson et al [9] define social norms as “(i) shared (and thereby social), (ii) expectations on behaviour by (iii) groups.” Social norms form between individuals, groups, and institutions; those are important to observe to understand the inclusion and exclusion of various stakeholders. Social norms are therefore similar to legal norms in that they create expectations on certain groups. The difference is that social norms are local and not regulated by the State. We now report on social norms regarding young carers and AI research.

### The Difficulties of Identifying Young Carers

While the section on legal norms demonstrated that there are legal frameworks in place for young carers in the United Kingdom, the reality is that there are difficulties implementing the laws both on a systematic and individual level. This results in nonidentified young carers falling through the nets of identification, assessments, support, and protection. Leu and Becker [2] set out the following three reasons why laws do not have the intended consequences: (1) due to the complex landscape of laws in the United Kingdom, whereby most professionals are not aware of specific legal requirements and responsibilities placed on them to identify and assess; (2) there is a gap between the ambition and the purpose of the law, with professionals within health care, education, and social work not implementing the law; and (3) there are insufficient resources to ensure professionals become aware of their legal responsibility [2]. The APPG report further illustrated the latter by mentioning that despite the rise in awareness, there are not enough resources to be able to undertake those assessments, which leads to more than 6 months waitlists for assessments [1]. This is further exacerbated by the term “young carer” not being well-defined in law or research [17,28], which also results in barriers for young people to recognize themselves as young carers [3]. Law can thus only go so far. Nevertheless, young carer scholars agree that laws are essential to legitimize young carers’ responsibilities which can help alleviate some of their care-related responsibilities, although laws are unlikely to fully eliminate these responsibilities [17].

Self-identification is a common challenge faced by informal caregivers generally. Carduff et al [84] pointed to the following three barriers to identifying informal caregivers: (1) taking care of one person is often a gradual process, and therefore carers do not identify as taking care of someone, but rather view it as part of their relationship to that person. This often leads to a health or social care professional making the person aware that they are a carer; (2) as the care receiver’s condition deteriorates and their needs become more demanding, the informal caregiver may struggle to prioritize their own well-being. In practice, this can result in them not seeking support or assistance with their caregiving responsibilities and instead continue doing so unsupported; and (3) there is a lack of clear pathways on when health care institutions should begin to support carers. This often leads to health care professionals being reactive rather than proactive [84]. Teachers also play a key role since young people under 16 years old in the United Kingdom must attend school. Yet, a study involving 800 teachers in the United Kingdom found that almost half of the teachers said that they would not feel confident that they could recognize a young carer; furthermore, the study also found that 57% of those teachers said that young carers will hide their situation from figures of authority [85]. Since 2022, the Department of Education in the United Kingdom requires schools to specifically report on numbers of young carers, although many schools still fail to record that group—with 56% of schools not reporting any this academic year (2025-2026) [43]. These types of data from UK health care institutions do not exist.

Although there are issues of identification stemming from institutions, identifying young people as carers might not have such an impact on their caring responsibilities. Alexander [86] found that laws in England helped shape young carers’ emotional lives and the significance of the care work they provide, but did not alter the arrangement of care work that they undertake. On the level of the individual, Aldridge et al [87] have pointed to the reluctance from families generally as well as children themselves disclosing that they are young carers, in part due to bullying and stigmatization toward the young carer and in part to the potential involvement of social services taking away the young carer. Stigma that a young person feels due to being a young carer is often mentioned as a barrier [88]. This “otherness” (ie, stigma) has been explored by researchers who found that this is either because of being a carer or by association to the person they care for, which led to young carers withdrawing and cutting themselves off from their social world [89]. There might also be a normalization of the care responsibilities the young person has [90]. Or there might simply not be enough services available to the person requiring care resulting in the young person filling that care void [90].

### Research Priorities in AI and Health Care

Social norms also exist within research communities. By highlighting these norms within AI for health care, we can better understand the need to challenge current research practices. It is evident that within the context of AI, young carers are overlooked and insufficiently included in the research focus. This might not be surprising when looking at how AI research communities are conceptualizing the challenges that lay ahead—a rapid change in the aging population impacting demographics [91,92] and the shortage this will likely cause on health care staff [93]. This narrative indicates a need in finding solutions to these issues, which AI researchers have found to be (unsurprisingly) technology solutions targeted at patients and health care staff. Consequently, the responses from the AI community to health care include technologies for early detection and diagnosis, to treatment, to prognosis evaluation (eg, study by Jiang et al [94]); or AI systems to assist older adults (eg, study by Qian et al [95]). With some studies pointing out that families are key to ensure the implementation of the technology in elderly care (eg, studies by Lee et al [96], Fotteler et al [97], and Kavčič et al [98]).

Generally, AI systems for health care mostly overlook children as well as ensuring that those are child-centered [99]. Although, some AI researchers are attempting to map out how to design AI systems for children, to ensure that those are age-appropriate [73]. However, to overcome this gap, Chng et al [99] offer recommendations, which include ensuring that AI systems in health care promote children’s development and well-being as well as must include children during the development process. Beyond frameworks, scholars have reported not being able to include children, such as siblings, during their data collection on the use of smartwatches by families with children with attention-deficit/hyperactivity disorder (ADHD) [100]. Perpetuating the invisibilization of young carers.

However, AI systems are *socio*technical, in that they are placed within a specific social context. Yet the anticipated use of AI systems will be steered by the framing of societal issues—often iterated by funding opportunities [12,13]. Meaning that AI systems impact social structures and organizations of the setting, even if this is not anticipated for. This is exemplified by a study looking at clinicians’ uses of AI systems in a clinical setting; Zając et al [101] split between the *technical* as (1) the training data and machine learning model, (2) system integration and data used, and (3) user interface, and the *social* as (1) users and system use, (2) workflow and organization, and (3) health care institution and political arenas. Although the authors note that the social and technical cannot be simply detached from one another and they are heavily dependent on the context, this will still have a significant impact on the workflow. This is also illustrated on robots for care settings; the current available technology is not currently fit for the purpose of caregiving fully autonomously, it requires setting up and monitoring by the care staff to function [102,103], and this burden will likely fall on informal caregivers too as States require longer independent living solutions [16] in part due to austerity [46,104]. The *socio*technical aspect of AI systems means that AI researchers must assess how technologies impact direct as well as indirect users, such as the perception and impact of assistive technologies on care receivers and informal caregivers—as these are already reported to differ, see for example, with robots for care [60,96].

### Takeaway on Social Norms

This section demonstrated the current difficulties of attending to young carers in society, and the direction AI research is heading toward. These are in stark contrast from one another; on the one hand, young carers are particularly difficult to identify throughout health care and education services; while on the other, AI systems aim to alleviate a care crisis due to change in demographics. Both issues should not be separated; they are part of the same coin. Young carers represent one side of the coin, who actively fill a shortage in health care services void by providing necessary and essential care; while AI systems represent the other side of the coin, which are being developed to prevent and/or assist in health care tasks and mainly including care receivers and health care professionals.

In practice, this means that the technical aspect of AI systems does not integrate possible solutions aimed for and at young carers currently. Yet, it is likely that once these AI systems are deployed, the social aspect of these technologies will become more apparent and will impact young carers—such as ensuring AI systems function. By not directly integrating young carers’ views or data points specific to young carers, we are likely to reproduce and amplify their current realities. Accordingly, this position paper underscores that overlooking young carers is not just a missed opportunity but a serious concern.

We subsequently demonstrate how the AI community can challenge the status quo and integrate young carers as specific users in AI systems.

## Strategies: How to Account for the Unaccounted

### Overview

Addressing young carers in AI research requires concrete interdisciplinary collaboration rather than a general appeal to domain experts. Relevant expertise includes AI and machine learning researchers, HCI and HRI specialists, clinical informatics researchers, pediatric and adolescent health professionals, education and pastoral care staff, social workers, safeguarding leads, young carer service practitioners, disability rights scholars, legal and data protection experts, ethicists, and young carers themselves. Each domain contributes a different form of necessary knowledge—technical researchers can identify how systems are designed and evaluated; educators and clinicians can explain where young carers become visible or remain hidden in practice; social care and safeguarding professionals can assess risks and referral pathways; legal and ethics experts can clarify duties around consent, data protection, and best interests; and young carers can explain what forms of support are acceptable, useful, or harmful in everyday life—especially relevant to capture norms.

Norms-in-the-loop’s framework [9] provides operational outcomes for young carers with regards to AI systems; whereby contextual, legal, and social norms will directly impact the (1) design, (2) datasets, and (3) in situ personalization; we sum those three up as follows:

1. Design: the developers decide who the application is for and how those will use it, but might miss out on key stakeholders or overlook essential needs. This will likely result in the reproduction of unfair norms through design.
2. Datasets: how the dataset is collected might overlook the context in which it was collected and might not fit the context it is used for. The dataset might collect complex social norms that are alleviating shortfalls of current legislation, or overlook discriminatory social structures, for example.
3. In situ personalization: this encapsulates ideas of personalization of a set application within a specific context (or situation). This notion may still rely on universalization and stereotypes to develop rather than contextualizing actual practices the system is attempting to assist with.

To make the framework practically applicable to young carers and AI research, Table 1 maps the 3 proposed strategy areas onto the design, dataset, and in situ personalization stages of norms-in-the-loop at different levels—institutional, care-receiver, and young carer level. This mapping shows that young carers can be overlooked or harmed at different points in the AI lifecycle, and that each proposed strategy requires different safeguards:

The strategies above do not apply equally to all forms of AI systems. Institutional identification and support strategies are most relevant to predictive analytics, digital triage, educational analytics, clinical decision support, identification of young carers, and information systems used by schools, health services, and social care [18,94,101,105]. Care receiver–focused strategies are most relevant to assistive technologies through smart home systems and monitoring technologies, with social robots [106,107], rehabilitation games [108], and personalized health care monitoring [95]. Young carer-directed strategies are most relevant to educational AI, information tools [52], peer-support platforms, well-being smartphone apps [54], conversational agents [53], and carefully governed social robots [7]. Distinguishing between these modalities is important because the ethical and practical risks differ; a school-facing risk raises different concerns from a young person’s optional well-being app, and a smart monitoring system in the home raises different concerns from a rehabilitation game used jointly by a sibling or/and care receiver.

**Table 1.** Abstract analytical mapping of proposed strategies to account for young carers within AI systems*.*

Strategy area	Design implications	Datasets implications	In situ personalization implications
Institutional systems	Young carers should be defined as a relevant stakeholder group in systems used by schools, health care, and social care. Systems should be designed to support sensitive professional judgment, not automated labeling.	Datasets should not rely only on visible or already identified young carers. Proxy indicators such as attendance, behavior, care receivers’ illness, or service use must be treated cautiously.	Alerts should trigger supportive human conversations, not automatic referral, punitive action, or covert disclosure.
Care receiver-focused systems	Systems designed for patients, people with disabilities, or older adults should account for the possibility that a child may be involved in care.	Data models should consider household caring arrangements without assuming that all carers are adults.	Deployment should reduce burdens on young carers rather than shifting technical monitoring, troubleshooting, or emotional labor onto them.
Young carer-directed systems	Young carers should be involved in defining acceptable support, including education, information, peer connection, and well-being tools.	Data collection should be minimal, age-appropriate, consent-aware, and sensitive to stigma and family privacy.	Tools should be low-burden, accessible, optional, and integrated with real support pathways rather than replacing human services.

### Operationalizing Norms-in-the-Loop

To operationalize norms-in-the-loop fully, clear data on young carers need to be reported and be part of research to understand what in situ personalization is desirable and possible. Consequently, for now, we call for States to first establish some legal basis that accounts directly for young carers (refer to Significance of Law for Young Carers section). This would allow for clear reporting and identification of young carers, also enabling researchers to include young carers in the development of AI systems. While this is ongoing, ethics boards must allow for research to be done with young carers—this is especially pertinent as researchers have reported on not being able to include siblings of children experiencing ADHD in a family setting for co-designing purposes [100].

To operationalize norms-in-the-loop currently, relevant example uses can be identified in clinical risk prediction, digital triage, or population health management systems designed to identify patients or households at risk of deterioration, unmet need, or avoidable service use [18,94,109]. Such systems commonly draw on structured data such as diagnoses, medication use, school attendance, service contacts, hospital admissions, missed appointments, safeguarding records, or social care involvement. If young carer status is absent from the dataset, inconsistently recorded, or treated only as a background family variable, the system may fail to identify a child whose educational attendance, mental health, or well-being is being shaped by hidden caregiving responsibilities [1,17,47,76]. Conversely, if the system infers caring status from indirect signals such as school absence, behavioral change, care receiver’s illness, or household service use, it may generate false positives, stigmatize families, or disclose a child’s caring role before the child has chosen to share it.

Concerns are that (1) at the design stage, developers may not define young carers as a relevant stakeholder or outcome group; (2) at the dataset stage, young carer status may be missing, inconsistently coded, or inferred from proxies that reflect institutional visibility rather than lived experience—especially since no country except the United Kingdom has clear regulations and reporting on young carers [2,3]; and (3) at the in situ personalization stage, alerts or recommendations may be interpreted by teachers, clinicians, or social workers in ways that either support the young person or increase and reproduce problematic social norms—such as stigma and family anxiety—as well as surveillance. The central issue is therefore not simply whether AI systems can identify young carers, but whether they can do so lawfully, proportionately, transparently (in line with legal norms [10,21,73,83]), and in ways that prioritize the child’s best interests and agency as well as the care receiver’s (in line with disability rights and young carer scholars [23,24]). The latter meaning that care receivers’ condition should not be pathologized and a voice must be given to care receivers, while also accounting for young carers in order to give adequate support to both.

### Strategies Based in Participatory Design

AI researchers must include young carers’ own voices when designing and/or evaluating the deployment of AI systems that will affect them—even if they are viewed as an indirect end user. This requires participation of young carers in research. Participatory design methods seek to mitigate adverse applications of AI systems through the inclusion of various stakeholders—especially from vulnerable populations in society [110]. Expertise is sought from an active partnership on how to attempt to solve an issue that will affect that community directly [111]. Furthermore, within young carer research, Joseph et al [17] identified participatory research as lacking. Indeed, Joseph et al, in their overview of young carer literature and possible future directions, are explicit that active engagement with young carers and their families is needed [17]. But this must follow a rights-based approach to ensure the child’s best interest is central and that their perspectives are heard during all relevant research stages [112].

Participatory design with young carers also brings ethical and practical challenges that should not be minimized. Young carers may already be under considerable time pressure and may feel responsible for protecting family privacy [7,88]. Involving them in research can therefore create additional burdens, particularly if participation requires travel, repeated workshops, emotional disclosure, or explanation of difficult family circumstances. Researchers must also navigate parental consent carefully. In some cases, parents or guardians may be unaware of the extent of the child’s caring role, may minimize it, or may be reluctant for it to be disclosed because of stigma or fear of State involvement (refer to Social Norms: The Gap Between Law and Society section and findings from the APPG report [1], as well as studies by Dinleyici et al [35], and Aldridge et al [87]). Participatory AI research with young carers should therefore be designed around safeguarding, flexibility, and low burden. This may include shorter sessions, choice of online or in-person participation, reimbursement, age-appropriate information sheets, independent support routes, clear limits to confidentiality, and careful planning around whether and how parental consent is obtained. The ethical aim is not simply to include young carers in design, but to ensure that inclusion does not reproduce the very pressures, invisibility, or lack of agency.

### Strategies at Institutional Level

At the institutional level, we include educational, medical, and social services institutions. All of these have a responsibility in the United Kingdom to screen for and identify potential young carers to then assess, as discussed in the Legal Norms: Laws for Young Carers and AI section. Having responsive institutions to aid with identifying young carers allows for more prevention of (excessive) care responsibilities on young people, as well as access help and support. Although this would need to be continuously reviewed to accommodate for children’s change in circumstance which can occur at different points in their lives, as well as adhere to legal obligations (refer to Legal Norms: Laws for Young Carers and AI section [81,82]). This also requires special attention to privacy concerns and how to address those sensitively—further discussed in contextual norms regarding the deployment of AI systems. Disregarding children’s care role leaves them unsupported and they fall through institutional gaps (a current issue pointed out by Nordenfors and Melander [64] when there are no regulations in place for young carers). However, overfocusing on young carers’ caring role may undermine family dynamics or obscure the fact that young carers are children and young people first [1]. Accordingly, interdisciplinary research with a whole-family approach is necessary [25].

Turning to how institutions could attend to young carers. First, within the educational setting, this could be achieved by directly accounting for a subgroup of young people who are young carers, either through monitoring attendance—such as predictive systems—or to help staff in education navigate how likely a child is a young carer—such as conversational agents. This would in part support educational staff to recognize potential young carers and help identify the young person, which is an ongoing reported issue [85]. Or potentially using educational analytics to flag students at risk of falling behind academically or socially due to hidden care roles (based on attendance, academic performance, and behavioral patterns). These alerts could trigger pastoral follow-ups or support referrals. Second, within medical institutions, they would likely come directly into contact with the care receiver more than with the informal caregiver, yet there would likely be an informal caregiver present (even if virtual through texting). Understanding the presence of informal caregivers could be nonintrusive, such as a check-in system integrating a question to the patient on whether anyone is accompanying them, or a health care professional making a note of it; in turn, this kickstarts an assessment process to help support informal caregivers before they feel overwhelmed with care responsibilities [84]. This would align with current AI system goals to provide comprehensive, personalized health care (eg, study by Taimoor and Rehman [109]). Overall, this would result in potential young carers being directed to relevant authorities for assessments. Finally, social services could partly rely on AI systems to conduct assessments to bring more stakeholders into it, and ensure a whole-family approach. Using such a tool would help understand different needs across families through more accessible and possibly wider participation in assessments of relatives and friends. This is especially pertinent as there is currently no standard assessment for young carers, including in the United Kingdom [88,113], but would allow for a comprehensive approach on how to support them.

However, AI-supported identification of young carers raises a significant ethical tension. As the social norms section discusses, young carers may actively conceal their caring role because of stigma, not knowing they are a young carer, or anxiety that disclosure could disrupt family life [36,85,87,90]. For this reason, AI systems should not be framed as tools for covertly detecting or outing hidden young carers. The goal should not be algorithmic surveillance of children [15], but the creation of proportionate, transparent, and supportive systems that help trusted professionals notice possible unmet needs and open sensitive conversations. AI systems could therefore provide resources or training for professionals on how to communicate with potential young carers effectively and ethically.

Any institutional use of AI in this context should therefore be governed by clear safeguards. These include child-centered explanations of how data are used, meaningful opportunities for young people to express their views, strict limits on automated decision-making, human review by trained professionals, careful consideration of false positives and false negatives, and pathways that offer support rather than punishment or family surveillance. Where a system flags possible caring-related needs, the response should be pastoral and supportive, not investigative by default. This distinction is central—AI systems may help institutions become more attentive to young carers, but they should not remove young carers’ agency over disclosure or intensify the monitoring of vulnerable families.

Finally, institutions should establish clear responsibility for data quality, system oversight, access controls, audit procedures, professional responses to alerts, and mechanisms through which children and families can question or correct information. Algorithmic errors may arise from incomplete, outdated, or inconsistently recorded data, the use of biased proxy variables, differences between the population on which a system was developed and the population in which it is deployed, or changes in family circumstances over time. False positives could wrongly label a child or family, potentially causing stigma or unnecessary intervention, while false negatives could reproduce young carers’ existing invisibility and prevent access to support. Consequently, AI-generated outputs should not independently determine safeguarding, educational, medical, or social care decisions; they should remain subject to review by appropriately trained professionals. Governance is therefore as important as predictive performance.

### Strategies for Care Receivers

Care receivers should not be treated as a uniform group. The implications of AI systems will vary substantially depending on who receives care, the nature of their condition, the relationship to the young carer, and the household context. A young person supporting a parent with a fluctuating mental health condition may face different risks from a young person helping an autistic sibling, a grandparent with dementia, a parent with substance use difficulties, or a family member with cancer or another progressive illness. These differences affect both the potential value and potential harms of AI systems. However, to this day, no research has been conducted on how AI systems can support young carers in these different constellations.

Care receivers and young carers have an established relationship, regardless of the reason the person is now in need of care. Hence, the critiques from the disability rights scholars on young carer services potentially undermining the role of the parent, even if the parent requires some assistance [22,23]. This keeps parents’ condition within medical parameters that do not account for the social aspect, where the parent does provide vital care to their children even if they are in need of assistance themselves [24]. This must be kept in mind by AI researchers to not overly pathologize the care receivers’ medical condition and also account for the social aspect of the condition [114]. In practice, this means being mindful of ongoing family and friendship dynamics, embedding the young person (also as a carer) within AI systems as those technologies are sociotechnical, even if the technology is targeted solely for the care receivers’ care needs. In turn, this identification could ensure that the child is not taking on excessive care responsibilities.

We can give various strategies; however, each is dependent on the issue care receivers would like help with that could also be of assistance to young carers. For example, reminder systems, fall detection, or medication monitoring may be useful in some contexts involving dementia, frailty, or physical illness, but may be less appropriate where the central issue is emotional distress, substance use, family conflict, stigma, or fear of external intervention (refer to The Difficulties of Identifying Young Carers section on the latter) [5]. Similarly, social robots or companion technologies [14] or multiplayer rehabilitation games [108,115,116] may reduce loneliness for some care receivers, but may also create maintenance burdens, privacy concerns, or unrealistic expectations within households already under financial and emotional strain, discussed more in the contextual norms section. By designing and understanding how to personalize applications directly with young people (the sibling, grandchild, friend, or child of the care receiver), it would allow for AI systems to help with care responsibilities in a fun way, and AI strategies for care receivers and young carers should therefore be tailored according to condition type, family relationship, risk profile, household resources, and the expressed preferences of both the care receiver and the young carer.

Another strategy may also be creating AI systems to help care receivers manage some of their care needs alone. This can be through systems recognizing that the care receiver may gain more independence [48], or getting emotional and psychological support, such as conversational agents [117] rather than potentially (solely) by the young carer. Although, as pointed out above, this should not pathologize the care receivers’ condition. Put differently, AI systems should ensure that they are continuously readapting to the individual’s needs [48], allowing the care receiver more independence and the ability to decide if they want an informal caregiver to be as present for caregiving purposes. This could subsequently support young people in their care role or even alleviate some care responsibilities and worries directly; for example, alerting informal caregivers if required—such as a fall or incorrect intake of medication. Understanding both care receivers’ perspective and informal caregivers’ and understanding how they envision AI systems to aid them is paramount, as various scholars have explicitly studied potential tensions and challenges that arise between the carer and the care receiver regarding care responsibilities [60,96], which might be amplified through the use of technologies if we do not directly account for the tensions in the design [58-60,118]. Consequently, the perspective of young carers would still need to be accounted for, to ensure that AI systems are suitable for the care receiver.

### Strategies for Young Carers

As this paper has set out, there are various difficulties and challenges young carers face; however, there is only 1 study to date that maps out in practical ways how AI systems could directly help young carers, with a focus on social robots [7]. Meaning that much needs to be done here, starting with young carers being named as stakeholders within ethics applications to do research with them. “Young carer” as a label is a very all-encompassing category; it can range from children providing care to a sibling who has a terminal illness to a parent who has an alcohol or substance use disorder, for example. Furthermore, it is unlikely that a young person providing care for a family member or a friend can be eradicated—especially with the ongoing and increasing difficulties of access to public services [46]. Therefore, research must also be directed at young persons that provide care, with a recognition that young carers represent a heterogeneous group. Studies from young carer researchers suggest that young carers want assistance with their care role, but also want assistance so that they can attend school as well as be part of their communities [7,76,119]. Current AI research developments could help young carers with self-disclosure around care burden [56], or sustain (and possibly create) these key social relations [120], or peer-support responses [53]—all currently being targeted at adult carers. Nevertheless, these mark potential avenues that are necessary to understand how to support young carers, so that they are more equipped regarding their care responsibilities yet also have more time for themselves.

The socioeconomic context of young carers must also shape any AI strategy. Since young carers are more likely to live in poverty [8], interventions that assume access to expensive devices, stable broadband, private space, digital literacy, or ongoing technical support risk widening rather than reducing inequalities [10]. Social robots, smart monitoring systems, and sensor-based technologies may be unrealistic or inappropriate for many households unless they are publicly funded, maintained by services, and designed around low-cost access. Even apparently simple digital technologies may exclude families with limited data plans, shared devices, unstable housing, language barriers, or low trust in statutory services. Nevertheless, AI systems are still being developed and must account for stakeholders those technologies will impact.

Furthermore, AI systems can also support young carers in their life as children and teens. For example, the APPG report highlights challenges young carers face around attending school, with a higher amount of absenteeism than average (on average, a young carer will miss 27 school days per academic year in the United Kingdom) as well as difficulties juggling care responsibilities with homework [1]. As AI systems in education continue to make headway [4], it is possible that there would be useful applications for young carers, either for supplementary education through tutoring or how to balance care responsibilities with schooling. To provide support, AI systems could be used within education or give relevant information to be given to young carers in a way that is suitable for young people and relevant to the care that they currently or will provide [7].

AI systems, however, should not be presented as a substitute for properly funded social care, educational support, respite, or young carer services. The most realistic near-term applications may be low-burden technologies embedded within existing institutions, such as school pastoral systems, young carer services, health systems information pathways, or local authority assessment processes. Where more advanced technologies are proposed, such as social robots or smart home monitoring, researchers and commissioners should explicitly address cost, maintenance, training, data governance, and who is responsible when systems fail. Without this, AI interventions may benefit only the most digitally included families while leaving the most disadvantaged young carers further behind.

### Takeaway on Strategies

This section demonstrated how to operationally account for young carers throughout AI systems’ life cycle, namely in design, datasets, and in situ personalization. We do so by zooming in on 3 proposed strategy areas—institutional, care receiver, and young carer levels. All 3 represent different concerns, such as (1) how to help medical staff identify young carers at the institutional level, (2) how to adapt to family dynamics at the care receiver level, and (3) how to best support young carers themselves while acknowledging various norms at play. This should ideally be achieved through participatory methods to include young carers and be proportionate, transparent, and supportive systems. We showcase concrete ways to account for young carers in Figure 3.

**Figure 3.**
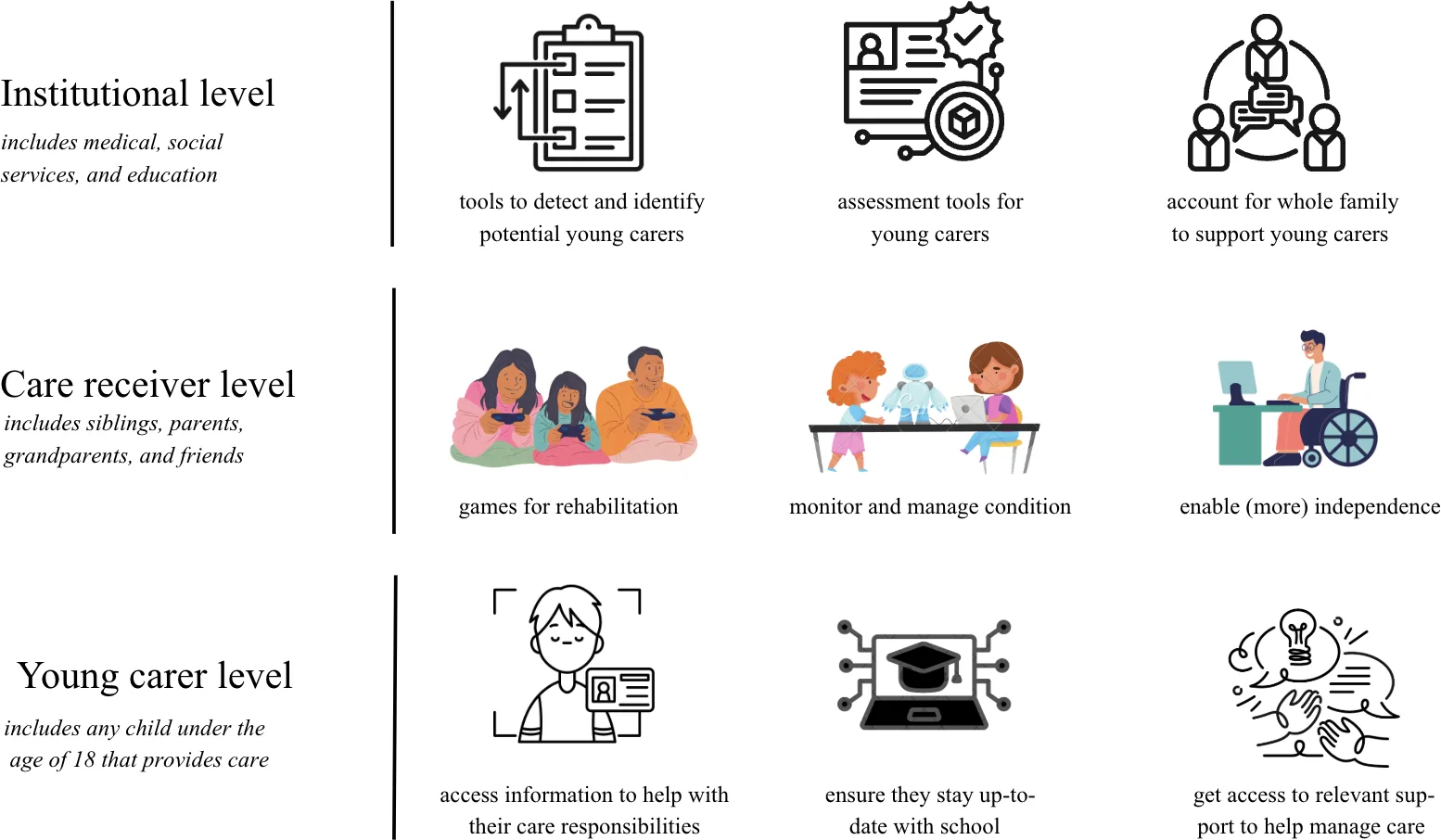
Illustrative examples to concretely account for young carers at the institutional level, care receiver level, and young carer level in AI systems. All should align with the United Nations Convention on the Rights of the Child (UNCRC), privacy laws, and recognize children as carers.

## Conclusion

This position paper marks how young carers are an overlooked stakeholder group in AI research. Yet, if young carers remain unrecognized in AI systems, this will reproduce existing institutional and societal blind spots. Accordingly, using the norms-in-the-loop framework by Larsson et al [9], we interwove young carers and AI systems within contextual, legal, and social norms, to in turn demonstrate how those are, or can be, embedded and reproduced in AI system’s design, datasets, and in situ personalization. We relied on the United Kingdom as a concrete case since it is the only country in the world to have specific laws on young carers [3], resulting in clear data on young carers [42,43] and demands on institutions for better identification and support for young carers [1,75,76]. Although this paper is extended beyond the UK context, as many researchers demand better support for young carers that spans across the world [2,3,17,24,26,29,30,119], which AI systems can help toward. The United Kingdom can thus be viewed as a template for how to help children that provide care and develop AI technologies that help them. Furthermore, it is most likely that current AI systems are already collecting data on young carers across institutions—especially health care, social services, and education—but are not recognizing these data as pertaining to young carers. The challenge is therefore to design AI systems that can recognize caring contexts without reducing young carers to risk profiles or removing their agency, which norms-in-the-loop attends to. If data on young carers are made visible through poorly governed inference or surveillance, AI may perpetuate and expose them to stigma, misclassification, or unwanted intervention. This paper thus presents how to concretely use norms-in-the-loop as a framework to attend to a vulnerable and overlooked stakeholder. Although, we urge AI researchers to view this framework as one that must be recalibrated for each envisioned AI system.

This position paper concludes that young carers should be accounted for across three connected areas including (1) institutional systems that may identify or support them; (2) care receiver-focused technologies that may indirectly affect them; and (3) young carer–directed technologies that may support their education, well-being, social connection, or access to information. Across all 3 areas, participatory design is necessary but not sufficient. It must be accompanied by safeguarding, children’s rights, child-centered consent processes, a whole-family approach, attention to poverty and digital exclusion, and clear governance over how data are collected, interpreted, and acted upon.

In 5‐10 years, success would mean that young carers are no longer absent from AI and digital health research involving informal care. It would mean that medical, educational, and social care technologies recognize the possibility of children’s caregiving roles without exposing children to unnecessary surveillance or harm [14,15,73]. The 2 most urgent research priorities are therefore: first, participatory and ethically governed research with young carers to identify acceptable AI-supported forms of support; and second, informatics research on how young carer status and caring contexts can be represented, protected, and acted upon within real-world systems. AI systems should not be used to replace public services for young carers, but it may help institutions and researchers better recognize, support, and protect a group that has too often been unaccounted for.

## Abbreviations

ADHD: attention-deficit/hyperactivity disorder
APPG: All-Party Parliamentary Group (in the Parliament of the United Kingdom)
DPA: Data Protection Act
EU: European Union
GDPR: General Data Protection Regulation
HCI: human-computer interaction
HRI: human-robot interaction
ICO: Information Commissioner’s Office
NEET: Not in Education, Employment or Training
UNCRC: United Nations Convention on the Rights of the Child
UNICEF: United Nations International Children’s Fund
